# A health-system-embedded deprescribing intervention targeting patients and providers to prevent falls in older adults (STOP-FALLS trial): study protocol for a pragmatic cluster-randomized controlled trial

**DOI:** 10.1186/s13063-023-07336-7

**Published:** 2023-05-11

**Authors:** Benjamin H. Balderson, Shelly L. Gray, Monica M. Fujii, Kanichi G. Nakata, Brian D. Williamson, Andrea J. Cook, Robert Wellman, Mary Kay Theis, Cara C. Lewis, Dustin Key, Elizabeth A. Phelan

**Affiliations:** 1 Kaiser Permanente Washington Health Research Institute, Seattle, USA; 2 University of Washington, Seattle, USA; 3 Kaiser Permanente Washington Health Research Institute, Fred Hutchinson Cancer Center, Seattle, USA; 4 Kaiser Permanente Washington Health Research Institute, University of Washington, Seattle, USA

**Keywords:** Falls, Deprescribing, Opioids, Sedative-hypnotics, Muscle relaxers, Antidepressant, Antihistamines

## Abstract

**Background:**

Central nervous system (CNS) active medications have been consistently linked to falls in older people. However, few randomized trials have evaluated whether CNS-active medication reduction reduces falls and fall-related injuries. The objective of the Reducing CNS-active Medications to Prevent Falls and Injuries in Older Adults (STOP-FALLS) trial is to test the effectiveness of a health-system-embedded deprescribing intervention focused on CNS-active medications on the incidence of medically treated falls among community-dwelling older adults.

**Methods:**

We will conduct a pragmatic, cluster-randomized, parallel-group, controlled clinical trial within Kaiser Permanente Washington to test the effectiveness of a 12-month deprescribing intervention consisting of (1) an educational brochure and self-care handouts mailed to older adults prescribed one or more CNS-active medications (aged 60 + : opioids, benzodiazepines and Z-drugs; aged 65 + : skeletal muscle relaxants, tricyclic antidepressants, and antihistamines) and (2) decision support for their primary health care providers. Outcomes are examined over 18–26 months post-intervention. The primary outcome is first incident (post-baseline) medically treated fall as determined from health plan data. Our sample size calculations ensure at least 80% power to detect a 20% reduction in the rate of medically treated falls for participants receiving care within the intervention (*n* = 9) versus usual care clinics (*n* = 9) assuming 18 months of follow-up. Secondary outcomes include medication discontinuation or dose reduction of any target medications. Safety outcomes include serious adverse drug withdrawal events, unintentional overdose, and death. We will also examine medication signetur fields for attempts to decrease medications. We will report factors affecting implementation of the intervention.

**Discussion:**

The STOP-FALLS trial will provide new information about whether a health-system-embedded deprescribing intervention that targets older participants and their primary care providers reduces medically treated falls and CNS-active medication use. Insights into factors affecting implementation will inform future research and healthcare organizations that may be interested in replicating the intervention.

**Trial registration:**

ClinicalTrial.gov NCT05689554. Registered on 18 January 2023, retrospectively registered.

**Supplementary Information:**

The online version contains supplementary material available at 10.1186/s13063-023-07336-7.

## Administrative information

Note: the numbers in curly brackets in this protocol refer to SPIRIT checklist item numbers. The order of the items has been modified to group similar items (see http://www.equator-network.org/reporting-guidelines/spirit-2013-statement-defining-standard-protocol-items-for-clinical-trials/).

**Table:** 

Title {1}	A health-system-embedded deprescribing intervention targeting patients and providers to prevent falls in older adults (STOP-FALLS trial): Study protocol for a pragmatic cluster-randomized controlled trial
Trial registration {2a and 2b}.	clinicaltrial.gov: NCT05689554, registered on 18 January 2023, retrospectively registered.
Protocol version {3}	At time of intervention launch KPWA IRB protocol version 17, approved December 20, 2020.
Funding {4}	This research is supported by the Centers for Disease Control and Prevention (Grant: 1 U01CE002967).
Author details {5a}	Benjamin H. Balderson*, Kaiser Permanente Washington Health Research Institute. (Benjamin.h.balderson@kp.org) Shelly L. Gray, University of Washington. Monica M. Fujii, Kaiser Permanente Washington Health Research Institute. Kanichi G. Nakata, Kaiser Permanente Washington Health Research Institute. Brian D. Williamson, Kaiser Permanente Washington Health Research Institute, Fred Hutchinson Cancer Center. Andrea J. Cook, Kaiser Permanente Washington Health Research Institute, University of Washington. Robert Wellman, Kaiser Permanente Washington Health Research Institute. Mary Kay Theis, Kaiser Permanente Washington Health Research Institute. Cara C. Lewis, Kaiser Permanente Washington Health Research Institute. Dustin Key, Kaiser Permanente Washington Health Research Institute. Elizabeth A. Phelan, University of Washington. ^*^ Corresponding author.
Name and contact information for the trial sponsor {5b}	Yara Haddad Centers for Disease Control and Prevention National Center for Injury Prevention and Control 1600 Clifton Road Atlanta, GA 30,333
Role of sponsor {5c}	The funder did not have a role in study design, data collection, management, interpretation or analysis, writing of the manuscript, or decision to submit the manuscript for publication. The funder did have regular contact with the research team regarding study progress.

## Introduction

### Background and rationale {6a}

Falls are the most frequent cause of fatal and non-fatal injuries among people aged 65 years and older [1]. Falls and their associated injuries have multiple serious adverse consequences—avoidable emergency department (ED) visits and hospitalizations, loss of independence, decline in physical function, nursing home placement, and reduced quality of life [2–5]. Of particular concern, national data from several countries indicate an alarming rise in fall-related ED visits [6], hospitalizations, and injury care costs over the last decade [7]. Thus, health systems approaches to prevent falls and fall injuries are urgently needed in order to “turn the tide.”

Medication use, particularly use of medications that affect the central nervous system (CNS), has been consistently linked to falls [8–10]. Common side effects of CNS-active medications include dizziness, sleepiness, and impaired balance and coordination. Use of CNS-active medications is common, with up to one quarter of older adults in the community taking at least one of these medications [11].

Practice guidelines recommend that prescribers review all medications with their older patients to minimize polypharmacy and the use of CNS-active and other high-risk medications [12]. However, this practice is not routinely followed [13–15] due to multiple barriers, including lack of healthcare provider and patient awareness that medications can cause falls [16], patients’ belief in the need for medication [17, 18], and provider reluctance to change prescriptions, even in the face of patients prompting the discussion [19].

The D-PRESCRIBE trial, a cluster-randomized trial in Canada delivered by community pharmacists, evaluated an educational intervention directed to patients and provider decision support. The intervention was highly effective in reducing use of potentially inappropriate medications by older adults, including CNS-active medications (benzodiazepines and non-benzodiazepine hypnotics) [20]. However, effects on health outcomes, including falls, were not reported. STOP-FALLS will adapt the D-PRESCRIBE intervention for use in an integrated healthcare delivery system in the USA and assess its effectiveness on medically treated falls.

### Objectives {7}

The primary objective of this trial is to test the effectiveness of a health-system-embedded deprescribing intervention on the incidence of medically treated falls with a sample of older adults who are long-term users of one or more CNS-active medications. CNS-active medication classes targeted by the intervention include the following: opioids, sedative-hypnotics (benzodiazepines and Z-drugs), skeletal muscle relaxants, tricyclic antidepressants, and first-generation antihistamines. Participants will be followed for up to 26 months. We will also examine (1) discontinuation or dose reductions of target medication (secondary outcomes); (2) serious adverse drug withdrawal events (ADWE), (3) unintentional overdose, and (4) death (safety outcomes); (5) evidence of planned dose reductions (process outcome), and (6) factors affecting intervention implementation.

### Trial design {8}

**Fig. 1:**
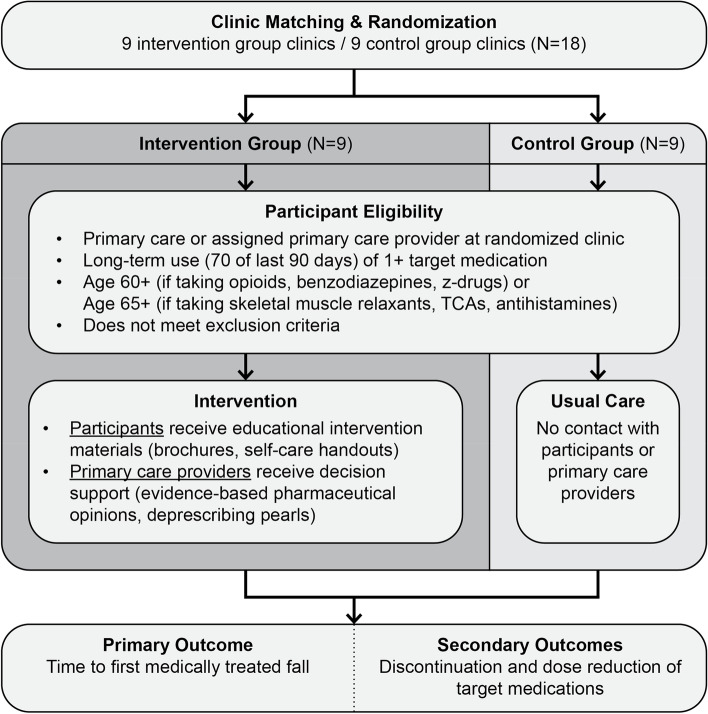
Study design for the STOP-FALLS cluster-randomized trial

This is a pragmatic, cluster-randomized, parallel-group, controlled clinical trial. In this trial, we are comparing the effectiveness of the intervention to a usual care control group on reducing rates of medically treated falls in older adults. The unit of randomization is the clinic, to avoid the risk of contamination if healthcare providers within a clinic were randomized (i.e., reducing the potential for intervention providers to communicate with control providers about the intervention and share materials). Eighteen clinics were identified for the trial, of which 9 were randomized to the intervention and 9 to usual care. Figure 1 illustrates the study design and flow.

## Methods: participants, interventions and outcomes

### Study setting {9}

Kaiser Permanente Washington (KPWA) is an integrated healthcare delivery system in Washington State that serves approximately 700,000 enrollees annually. About two-thirds of members are in the Integrated Group Practice (IGP), in which members receive KPWA insurance coverage and care from KPWA providers; the other one-third are insured by KPWA but see contracted providers outside the KPWA healthcare system. This trial is limited to enrollees of the IGP which allows for access to complete medical utilization data.

### Eligibility criteria {10}

#### Clinic eligibility

Of the 35 KPWA primary care clinics, we identified a subset of clinics to represent a diverse geographical range, excluding clinics that served a small eligible patient population to meet power calculation estimates for participant sample size while minimizing the number of clinics randomized. We enrolled 18 KPWA primary care clinics to participate in the trial. The 18 clinics were then matched by geographic location and size, creating matched clinic pairs, in which we randomized the matched clinic pairs to either the intervention or usual care arm, yielding 9 clinics randomized to intervention and 9 to usual care (see Sequence Generation section for details).

#### Participant eligibility within clinic

The study participant sample for each intervention and usual care matched clinic pair is identified at the time the intervention is implemented at the intervention clinic. For opioid and sedative-hypnotic medications, eligible participants are aged 60 years or older, while for skeletal muscle relaxants, tricyclic antidepressants, and first-generation antihistamines, eligible participants are aged 65 years or older. Eligible participants must be long-term users of the target medication, defined as pharmacy dispensing of at least one of the target medication classes for at least 70 of the prior 90 days. Further, eligible participants must either be assigned to a primary care provider (PCP) or have had 1 + visits in the prior year with a PCP at one of the 18 KPWA clinics participating in the study. Once a participant is determined as having received care or having a PCP at a given clinic, their clinic assignment is fixed, and therefore their randomization assignment will be static throughout the study.

Individuals will be excluded for any one of the following reasons, all ascertained pragmatically (i.e., from electronic data sources): (a) diagnosis of dementia or a prescription for a medication used to treat dementia (i.e., a cholinesterase inhibitor or memantine); (b) residence in a skilled nursing facility; (c) metastatic cancer diagnosis in the prior 12 months; (d) receiving hospice or palliative care; (e) legally blind (unable to read print materials); (f) indication the participant requires a translator (cannot read materials printed in English); (g) enrolled in other KPWA opioid deprescribing research studies; (h) enrolled in a KPWA pharmacy-driven initiative to reduce opioid dose; or (i) diagnosed with opioid use disorder.

### Who will take informed consent? {26a}

The study is approved by the KPWA Institutional Review Board (IRB). Due to the pragmatic and educational nature of the intervention, the KPWA IRB granted a waiver of informed consent for eligible participants consistent with the requirements outlined in 45 CFR 46.116 Part F.3.

Intervention participants are mailed an invitation letter and information sheet informing them of their enrolment in the study because they may be taking a medication that increases fall risk. They are informed that participation in the research study does not affect their insurance coverage. Their PCPs receive a staff message via the electronic medical record (EMR), alerting them of their patient’s enrollment in the study, including general information on the study and next steps, and that they are under no obligation to act on any of the research materials or alter medical care in any way. All materials and communications to both participants and PCPs include information on how to contact the study team by email or telephone.

### Additional consent provisions for collection and use of participant data and biological specimens {26b}

Not applicable. We are not collecting any biological specimens or other materials requiring additional consent provisions.

## Interventions

### Explanation for the choice of comparators {6b}

The comparator for this study is usual care in nine matched control clinics. Usual care is a common comparator for pragmatic trials, as it captures a wide, realistic range of practice scenarios and controls for changes that may occur within the cohort and the healthcare system [21]. Given there is no contact with the usual care group (i.e., no participant survey or measurement), this further helps to create a “real-world” comparison.

Identical procedures will be used for mailing brochures to participants in the matched clinic pairs to ensure comparable rollout and address potential temporal changes within the delivery system. Specifically, at the time an intervention clinic starts the intervention, we will implement the same procedure set-up as outlined in the “Intervention delivery” subsection (below) for the matched usual care control clinic to define a participant’s study enrollment and outcome follow-up time.

### Intervention description {11a}

The STOP-FALLS intervention consists of two major components: patient education and provider decision support.

#### Patient education

Patient education consists of educational brochures and self-care/symptom management handouts. Educational brochures were adapted from prior deprescribing trials conducted in Canada for three of the medication classes targeted by STOP-FALLS: opioids, sedative-hypnotics (benzodiazepines and Z-drugs), and first-generation antihistamines [20, 22]. We adapted these materials with input from KPWA delivery system members, including clinical and pharmacy leadership and PCPs. The sedative-hypnotic and antihistamine brochures had been originally designed for older adults. The opioid brochure had been developed for a general population; we extensively revised it to focus on older adults and safety concerns in concordance with KPWA pain management guidelines. The study investigators developed new brochures for skeletal muscle relaxants and tricyclic antidepressants, as no pre-existing materials were available, and modeled them after those from the Canadian deprescribing trials. Patient input was obtained on the opioid, skeletal muscle relaxant, and tricyclic antidepressant brochures through a series of focus groups conducted with KPWA enrollees representative of our target study sample.

Self-care/symptom management handouts were created by the study psychologist (BB) for each of the following symptoms for which a target medication is often prescribed: anxiety, chronic pain, insomnia, and allergies. They emphasize non-pharmacological strategies and describe resources for managing symptoms. Handouts covering the relevant symptom(s) are mailed along with an educational brochure. In addition, the Centers for Disease Control and Prevention’s (CDC) “What YOU Can Do to Prevent Falls” pamphlet is included with each mailing; the rationale for this pamphlet is to highlight that there are several actions that can be taken to reduce the risk of falls, so that even if a participant does make any changes to their medications, they may take other steps to prevent falls.

#### Provider decision support

Provider decision support consists of two elements: An evidence-based pharmaceutical opinion (EBPO) and “deprescribing pearls”. The EBPOs, modeled after those of the D-PRESCRIBE trial [20], describe the risks associated with the target medication class, alternative evidence-based treatments that could be tried to help a participant reduce their use of the medication, and hyperlinks to practice supports for deprescribing (e.g., pharmacy consultation, mental health referral, and self-care support tools). Prior to intervention implementation, each clinic received a 30-min presentation on the study methods and patient and provider materials, with an emphasis that changes to medication prescriptions were up to their clinical discretion.

In addition to EBPOs, “deprescribing pearls” will be distributed to all intervention clinic PCPs, regardless of whether they have a participant enrolled. Providers in other settings have endorsed the need for guidance on how to initiate deprescribing discussions [23]. The content of the pearls was developed by STOP-FALLS investigators based on the published literature [24–26]. Each pearl also gives several examples of how to broach discussions of deprescribing with patients, referred to as “conversation starters.” The pearls were modeled after “clinical pearls” used by KPWA to disseminate clinical information updates and thus are anticipated to feel familiar to providers. Table 1 lists the topics for the thirteen pearls.

**Table 1:** Deprescribing pearl topics sent to primary care providers at all intervention clinics

	**“Deprescribing Pearl” Topics**
1	Medicines linked to falls
2	Sedative-hypnotics
3	Opioids
4	Over-the-counter (OTC) sleep aids
5	Skeletal muscle relaxants
6	Tricyclic antidepressants
7	Managing benzodiazepine and Z-drug withdrawal symptoms
8	Fight prescribing inertia
9	Pursuing opportunities for opioid deprescribing
10	Deprescribing and the patient-provider relationship
11	Return of symptoms from underlying condition
12	Deprescribing triggers
13	Deprescribing OTC antihistamines

All participant and provider materials have been carefully cross-referenced with KPWA clinical practice guidelines and reviewed by leaders in the KPWA delivery system so that all information and recommendations are concordant with KPWA guidance.

### Pilot testing

Study procedures and intervention materials were pilot-tested within a single intervention clinic (*N* = 142) and matched control clinic (*N* = 160). Participants in the pilot were excluded from the main trial. The pilot-tested study procedures for identifying patients via the EMR, sending mailed materials to participants and faxing decision support to providers. A clinician champion provided feedback to the site principal investigator (BB) on intervention acceptability from the clinic and provider perspective. Key points conveyed included an appreciation for the focus on deprescribing, a minor concern for the intervention generating additional clinic visits as a result of patients receiving intervention materials, and a strong preference for transmission of provider decision support via secure messaging in the EMR rather than fax. The pilot also determined that participants who received an opioid brochure had higher rates of declining to receive further mailings and have their healthcare utilization and pharmacy data used for research purposes. As a result of the latter finding, the study team sought and received IRB approval for a waiver of consent for identifying participants on the premise that differential refusals would bias a pragmatic trial. Participants in the pilot were excluded from the main trial.

#### Intervention delivery

For each intervention clinic, materials are mailed to a subset (approximately one-third) of eligible participants at intervals (mailing “waves”) to minimize burden on the healthcare system that might otherwise result from a large volume of requests from participants for appointments to discuss study materials with their PCP. For each wave, participants who have upcoming visits with their PCP are prioritized, then participants *without* an upcoming visit are selected to ensure that the target sample size for the trial is achieved. If a participant is identified as having a prescription for more than one of the target medication classes, they are mailed a corresponding brochure at least 90 days after the mailing of the prior brochure. In these cases, brochures are mailed in the following order: opioids, sedative-hypnotics, skeletal muscle relaxants, tricyclic antidepressants, and/or antihistamines. In light of prior research (Benjamin Balderson, personal communication) with KPWA’s older enrollees demonstrating high utilization of over-the-counter, first-generation antihistamines (i.e., non-prescription antihistamines), all participants will receive an antihistamine brochure regardless of whether they have a KPWA pharmacy record of an antihistamine prescription.

Providers will receive a staff message via the EMR, synchronous with a brochure being mailed to a participant, that identifies that participant by name and gives the target medication class of the brochure that they were mailed. The staff message will include a hyperlink to the STOP-FALLS study website where the complete EBPO pertaining to that target medication class can be found.

Each deprescribing pearl will be sent in an e-mail to the clinic chief or other identified “clinical champion” at 2-week intervals. The recipient will distribute and promote the information in ways that are appropriate and consistent with how information is typically delivered at that clinic (e.g., emails, weekly meetings, daily huddles, posts).

#### Acceptability of intervention

To evaluate the acceptability of the intervention, 30 days after each medication brochure mailing date, intervention participants will be mailed a brief postcard questionnaire asking which brochure they received, how useful the information was, and how likely it is that they will have a conversation with their provider about their medication. Response to the questionnaire is voluntary, and data are collected anonymously. No other direct contact with participants for data collection will occur.

### Criteria for discontinuing or modifying allocated interventions {11b}

Participants may request to not receive subsequent mailed intervention materials. Otherwise, participants are considered enrolled and their healthcare utilization and prescription data will be included in analyses.

### Strategies to improve adherence to interventions {11c}

The intervention encourages but does not require behavior change on the part of the participant or their PCP. However, participant and PCP communication about the material and medication changes may be considered proxy responses to the intervention. We will closely examine medication prescriptions and instructions for discontinuation and tapering within the medical chart. We will also examine postcards returned by patient participants regarding if they intend to discuss materials with their PCP. See “Statistical analysis” for details.

### Relevant concomitant care permitted or prohibited during the trial {11d}

All routine and necessary medical care is permitted during the trial. Although the intervention materials encourage participants to talk with their PCP about their medication regimens, changes to medications are at the discretion of the participant and their PCP.

### Provisions for post-trial care {30}

Given the study does not require or restrict any medical care, there are no provisions for post-trial care.

### Outcomes {12}

#### Primary outcome

The primary outcome is a participant’s first (incident) medically treated fall post-baseline, where baseline is defined as the time point after study enrollment at which a first brochure is mailed (or proxy mailed for usual care participants) (see “Participant timeline {13}” section below for details). Medically treated falls will be identified from International Classification of Diseases, Tenth Revision (ICD-10) injury (S or T) codes or musculoskeletal diseases (M) code or fall-related cause of injury (W) codes associated with hospitalizations, emergency department visits, urgent care visits, and primary and specialty care visits. We will exclude injury codes with an associated motor vehicle crash code as the cause of injury, provided these codes are recorded within a 3-day window of each other. The ICD-10 codes for the primary outcome are provided in Additional File 1. An incident fall is defined using a 3-month washout period after the last pre-baseline fall if any occurred, to ensure that treatment is for an incident fall event.

#### Secondary outcomes

**Fig. 2:**
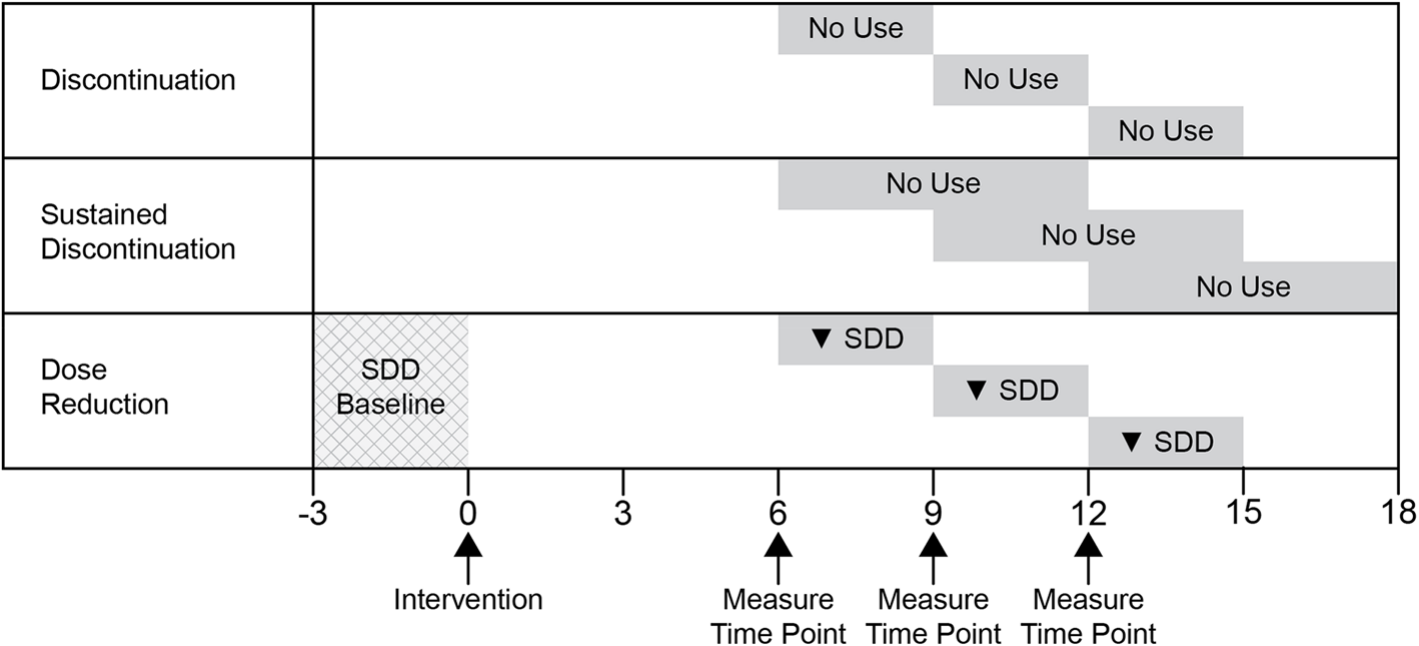
Definition of medication outcomes at primary (6 months) and additional timepoints (9 and 12 months) {12}

We will examine three medication outcomes measured at the level of the participant: discontinuation, sustained discontinuation, and dose reduction of any target medication (Fig. 2). Exposure to target medications will be obtained from computerized KPWA pharmacy files, which include drug name, dosage form, strength, amount dispensed, and number of days’ supply. To summarize over a medication class, we will operationalize dosage as an average standardized daily dose (SDD) based on methodology used previously (see Additional File 2) [27–29]. We will use morphine equivalents to standardize across opioids. For each target medication, we define discontinuation at a given timepoint as having no medications (SDD = 0) across 90 days following that specified timepoint. Sustained discontinuation at a given timepoint is defined as having no medications (SDD = 0) across 180 days following the specified timepoint. Dose reduction at a given time point is defined by taking the difference between the SDD in the 90 days prior to first brochure mailing date (average baseline dose) and the 90 days following the specified timepoint (Fig. 2). We use the target medication mailing date as the start of follow-up (or proxy mailing date for usual care clinics). For a participant prescribed multiple target medications who will thus receive multiple brochures (e.g., an opioid and benzodiazepine brochure), when examining the second medication discontinuation or reduction, baseline dose is defined at the time of second medication brochure mailing date, not first brochure mailed. We will further consider the three medication outcomes summarized across all target medication classes. Overall discontinuation, sustained discontinuation, and dose reduction are defined as discontinuation, sustained discontinuation, and dose reduction of the medication class targeted by the first mailed medication brochure, respectively. We consider these overall medication outcomes to be the main secondary outcomes.

#### Safety outcomes

Serious adverse drug withdrawal events (ADWE) due to opioids or benzodiazepines.

Urgent care, ED visits, or hospitalizations for an adverse drug withdrawal is the trial’s main safety outcome. For participants mailed an opioid or benzodiazepine brochure, we will identify possible withdrawal events using ICD-10 codes for drug (opioid or benzodiazepine) withdrawal and withdrawal symptoms (e.g., for opioids, nausea, diarrhea, abdominal pain), over 12 months from brochure mailing. Information related to the reason for the healthcare event and course of care will be abstracted from the EMR. Two study team members will independently review the abstracted chart, blinded to the intervention status and use a published algorithm to assess the probability that the symptoms represent an ADWE [30]. A third investigator will adjudicate disagreements.

##### Unintentional overdose

Reduction in use of CNS-active medications may result in fewer unintentional overdoses. We will examine ICD-10 Clinical Modification codes for overdose due to medications using a modified set of CDC codes for overdose [31, 32]. These codes are as follows: opioids T40.2X-T40.6X*; benzodiazepines T42.4X*; Z-drugs T42.6X*; tricyclic antidepressants T43.01*; skeletal muscle relaxants T48.1*; antihistamines T45.0X1A*.

##### Deaths

Deaths will be tracked for both study arms using death data in the KPWA automated data files.

#### Other measures

Provider plan for tapering or dose reduction based on prescription information. To supplement the medication outcomes as described above, we will also look for evidence of a plan for dose reduction (i.e., taper plan) from the medication instructions included with the prescriptions of target medications (the “signetur” or “SIG” portion of the prescription, a free-text field in the electronic health record). We adapted an algorithm to determine the most reliable indications of a provider starting a taper or discontinuation of a target medication [33]. We used the following terms: "taper", "decrease", "reduce", "lower", "wean", "cut down". Initially, we included "discontinue" and "stop" but after chart review found this led to false positives. Two investigators will independently review further data from prescriptions that include these terms, and a third investigator will adjudicate any disagreements that may arise [33].

##### Classification of clinics by deprescribing performance

As part of our implementation analysis, we will classify clinics as low, moderate, or high performers on deprescribing, characterized as the number of participants for which clinically meaningful deprescribing occurred divided by the number of eligible participants. Clinically meaningful will be defined as discontinuation, sustained discontinuation, or dose reduction at the 6-month timepoint.

### Participant timeline {13}

Primary care clinics are randomized, and then potential participants identified as having their PCP within the clinic. Identification of potential participants, and the start of enrollment and intervention delivery, occur on a rolling basis, with an intervention launch date scheduled for each intervention and control clinic pair. All potential participants meeting eligibility criteria for a given clinic pair are identified on the launch date, starting the 12-month intervention period for that clinic pair. As described above (“Intervention delivery”), only a subset (approximately one-third) of potential participants at each intervention clinic are enrolled and mailed study materials on the intervention launch date. Mail date (analogous to proxy mail date for usual care clinics) is the date on which a participant is enrolled in the study and follow-up for outcome data collection begins. Potential participants identified on the launch date but who become ineligible before enrollment could occur, and who remain ineligible for the duration of the 12-month intervention period, are not included in the study sample, since they are never mailed an intervention brochure. After completion of the 12-month intervention period for each clinic pair, participants are followed for individual-level outcomes until the end of the study period, with up to 26 months of follow-up. No further participant contact occurs following the 12-month intervention period.

Intervention launch occurs for 2–3 intervention/control clinic pairs per month. The study team mailed brochures to the first eligible participants on April 1, 2021. The last opportunity for enrolling and contacting participants occurred on June 16, 2022, and participant follow-up is scheduled to close on June 16, 2023.

### Sample size {14}

Our sample size calculations for the number of clinics to randomize were designed to ensure at least 80% power to detect a 20% reduction in the hazard rate of medically treated falls between the intervention and usual care clinics over 18 months of follow-up. Given the rollout to clinics over time, follow-up is expected to range from 12 to 26 months. For the sample size calculations, we chose the midpoint, i.e., 18 months of follow-up.

Sample size calculations were informed by estimates obtained from data on a historical cohort of potentially eligible participants at participating KPWA clinics. This cohort consisted of KPWA members meeting the study eligibility criteria as of January 1, 2018, and included follow-up for medically treated falls for 18 months. The 18-month proportion of medically treated falls was estimated to be 29%, and the intraclass correlation (ICC) was estimated to be <  < 0.001. We used several approaches [34] to calculate the ICC, and all approaches gave close to a zero ICC; to be conservative, we assumed a 0.001 ICC for our sample size calculations.

To obtain an estimate of the number of potentially eligible participants at each clinic, we pulled additional data on a cohort of eligible members as of January 16, 2020. These data were used to calculate an average cluster size of 183 individuals. To account for variable clinic sizes, we used the smaller harmonic mean cluster size of 154 individuals in the calculation of study power [35]. Further, for the purpose of calculating power, the outcome was assumed to be binary, i.e., at least one medically treated fall, as opposed to the time to first medically treated fall, which will be used as the outcome in the primary analysis. We expect minimal censoring in our data (5% annually), in which case the minor simplification of estimating the relative risk based on a binary outcome and inflating the sample size by 15% should be similar to estimating the marginal hazard ratio and is conservative [36]. Therefore, this sample size calculation closely mimics our approach given the underlying assumptions going into the marginal hazard model with robust standard errors that we will be applying.

Given the estimates above and their accompanying assumptions, it was determined that randomizing nine clinics to each study arm would provide 89% power to detect a 20% reduction (RR = 0.80) in the rate of medically treated falls in the intervention group compared to the usual care group. Power calculations were done using PASS 2019 software Version 19.0.1 [37] using a test for two proportions in a cluster-randomized design [38].

### Recruitment {15}

Eligible participants are identified by the study programmer using KPWA automated data. Participants receiving care in clinics assigned to the intervention group are thereafter mailed a notification letter about the research study occurring in their clinic, along with a brochure and self-help handouts.

## Assignment of interventions: allocation

### Sequence generation {16a}

To accommodate regional rollout of the intervention with concordant timing of study initiation between the intervention and control clinics, and to avoid large differences in potential sample size between intervention and control clinics, nine clinic pairs were identified based on geographic location and the number of eligible KPWA members receiving care. Clinics were then randomized to intervention or control using constrained randomization [39]. Using R software version 3.6.1, the study biostatistician implemented the randomization by considering all 256 possible arrangements of the eight clinic pairs and with one clinic in each pair assigned to intervention and one to control. Using estimates of the number of eligible members at each clinic, the difference in the average clinic size between the intervention and control groups was estimated for each possible arrangement, and the 10% with the smallest average difference in cluster size between intervention and control were retained. The final assignment was then selected at random from the remaining possibilities. To meet projected numbers needed, an additional pair of clinics was added after primary randomization was conducted. The intervention was randomly assigned within the additional pair.

### Concealment mechanism {16b}

The randomization occurred after all clinics had agreed to participate, all clinics were randomized at one time, and clinics were unaware of other clinics’ randomization assignment; therefore, randomization was concealed.

### Implementation {16c}

After the 18 clinics were selected and the intervention clinics recruited to participate, we obtained data on each clinic’s geographic region and eligible study sample size (described in the sample size section). From these data, we determined the 9 clinic pairs, and the study biostatistician generated the allocation sequence using R and shared the final randomization with the study team.

## Assignment of interventions: blinding

### Who will be blinded {17a}

Due to the cluster-randomized trial design with the level of intervention being the clinic, intervention clinic directors, providers, and staff are not blinded to randomization assignment. Participants within the intervention clinic are not blinded, but usual care participants are blinded. Participants’ postcard questionnaires will be anonymous and thus not linked to a specific participant. The source of outcomes data will be the EMR (e.g., medically treated falls, pharmacy data). The study programmers who will extract these data will not be blinded given they have access to the clinic information, but the same code for outcome assessment indexed by mailing date will be run once without clinic identifiers. The data will be coded so that others on the study team will be blinded until the final datasets are completed. The biostatisticians are not blinded but will not have access to follow-up outcome data until after final analytic datasets are complete, and the statistical plan will be finalized prior to receiving any follow-up data.

### Procedure for unblinding if needed {17b}

There is no foreseen need to unblind the study staff regarding outcomes data until the analyses are completed. If such a need arises, a second study programmer who is unblinded will consult with one of the investigators (BB) revealing minimal information to address the concern, and that investigator will be removed from any analysis meetings that might be influenced by this unblinding.

## Data collection and management

### Plans for assessment and collection of outcomes {18a}

Medically treated falls, medication prescriptions, ADWE, and healthcare utilization will be ascertained via the KPWA EMR and virtual data warehouse. Death and disenrollment are ascertained via the KPWA virtual data warehouse. See “Outcomes” section above for details on primary and secondary outcomes. As previously described, participants are sent a postcard regarding which brochure they received and if they intend to discuss the material with their PCP. These are returned voluntarily and anonymously and therefore are not linked to the participants’ medical record.

### Plans to promote participant retention and complete follow-up {18b}

The study has a waiver of consent; therefore, all participants are enrolled for outcomes data collection. No reminders are sent to participants regarding the postcard survey; response is completely voluntary.

### Data management {19}

Postcard questionnaires will be in hard-copy (paper) format and returned anonymously to the study team. Hard copies of the questionnaires are stored in locked files cabinets in KPWA research offices after the data are entered into KP computers. Participant data will be collected from KPWA electronic data sources by KPWA programmers. Participant data will be stored on a HIPAA-compliant secure server hosted, managed, and monitored by the Kaiser Permanente Washington Research Institute, with daily backups, and will be deidentified at the earliest possible opportunity. The linking file will be destroyed, per IRB guidelines, 5 years post study end date. Data management for the trial and details on their processes and procedures are specified in the Data Management Plan which is available from the corresponding author on request.

### Confidentiality {27}

Research data will be stored on password-protected computers on a secure server. Access will be restricted to staff using this information to perform study-related activities. Participants will be assigned a unique identification number; the file linking these numbers to personal identifiers will be stored separately from analytic data files. Data tables with any identifiers needed for mailing intervention materials to study participants (i.e., name, address) will be maintained separately from all other study data tables. All data files will be password protected. All employees at KPWHRI routinely sign a confidentiality form that covers access to all data encountered. Postcard questionnaire data are collected anonymously. No personal identifiers will be reported in publications or presentations.

### Plans for collection, laboratory evaluation, and storage of biological specimens for genetic or molecular analysis in this trial/future use {33}

Not applicable. We are not collecting, evaluating, or storing any biological specimens.

## Statistical methods

### Statistical methods for primary and secondary outcomes {20a}

Descriptive statistics will be computed, and appropriate graphical summaries (e.g., histograms, boxplots, scatterplots) will be generated for all variables across the intervention and control clinics to assess the comparability of baseline characteristics and follow-up times of each group. Although we matched and randomized on size and location of clinics and expect the KPWA population to stay relatively stable over the study time period, any participant characteristics that differ between groups at baseline, and which are known to be related to the outcome or likelihood of disenrollment from the health plan, will be adjusted for in analyses. We will include geographic region of the participant’s clinic, age, sex, and an indicator of prior falls in the adjusted models. Statistical significance will be indicated by a *P*-value < 0.05, and all tests and confidence intervals will be two-sided.

#### Primary outcome

We will use a time-to-event approach to compare time to first incident medically treated fall between the intervention and usual care groups. This approach accounts for censoring due to disenrollment from the health plan. Since death precludes the observation of a fall, we will use methods that account for competing risks. In this analysis, we consider non-fall deaths to be a competing risk; deaths that are contemporaneous with a fall event are considered to be medically treated fall outcomes. The statistical literature suggests that using multiple, complementary analysis approaches in competing-risk settings can provide richer information about any effects of an intervention on the outcome of interest and the competing risk [40, 41]. We will use robust standard error estimates in all analyses to account for correlation due to cluster randomization and all analyses will include an indicator for intervention and usual care clinic and all adjustment variables.

Follow-up time for all primary outcome analyses is defined relative to the first brochure mailing date for study participants in the intervention group and proxy first brochure mailing date for those in the usual care group (see “Participant timeline {13}” section above for details). The observed outcome for each participant will include the observation time (the earliest occurrence of first incident medically treated fall, death, disenrollment from the health plan, or the end of the study period), and indicators of whether an incident medically treated fall, death, or disenrollment from the health plan were observed.

Our primary analysis is to fit a cause-specific proportional hazards regression model for time to first incident medically treated fall accounting for the competing risk of non-fall death by censoring at the time of non-fall death. We will estimate an adjusted cause-specific hazard ratio for intervention effect and a 95% confidence interval and *p*-value [42–44]. Under this model, we can interpret the hazard ratio as comparing the instantaneous risk of a first incident medically treated fall among those who have not fallen nor died at any given time point between participants in the intervention and usual care groups.

To support this primary analysis, we will fit two further models. The first is a cause-specific proportional hazards regression model for time to non-fall death accounting for the competing risk of incident falls by censoring. Under this model, we can interpret the hazard ratio as comparing the instantaneous risk of death among those who have not fallen nor died at any given time point between participants in the intervention and usual care groups. We will next use a subdistribution hazards model to estimate adjusted cause-specific cumulative incidences of both first medically treated fall and death over time [45, 46]. We will display the adjusted cumulative incidence functions graphically. To explore timing of when the intervention may have occurred, we will conduct secondary analyses by comparing the estimated cumulative incidence in the intervention and control arms at 6, 9, and 12 months. We will provide 95% clinic-level bootstrap percentile intervals for the cumulative incidence at these time points.

Finally, we will run a secondary analysis using a composite outcome of time to first incident medically treated fall or death and fit a Cox proportional hazards model [40, 44]. We will estimate the hazard ratio and a 95% confidence interval. Under this model, we can interpret the hazard ratio as comparing the instantaneous risk of a first incident medically treated fall or death among those who have neither fallen nor died at any given time point between participants in the intervention and usual care groups. We will also estimate and display graphically Kaplan–Meier estimators of the probability of neither falling nor dying in both groups, and Nelson-Aalen estimators of the cumulative incidence of falls or death in both groups [43].

#### Secondary outcomes

We will examine three medication outcomes measured at the level of the participant: discontinuation, sustained discontinuation, and dose reduction of any target medication and of each target medication (Fig. 2). The primary time point for all medication analyses is the 6-month time point while 9 and 12 months are secondary time points. To investigate the effectiveness of the intervention on discontinuation and sustained discontinuation (both overall and target-medication-specific), we will fit a Poisson regression model with an indicator of study arm and all other adjustment variables. To investigate the effectiveness of the intervention on dose reduction, we will fit a linear regression model with an indicator of study arm and all other adjustment variables. In these analyses, we will not model the competing risk of death, since we are primarily interested in short-term effectiveness (6 months) and so risk of either death or censoring is small (< 5%). We will include follow-up time as an offset term (in the Poisson regression models) or as a weight (in the linear regression model) to account for censoring due to disenrollment from the health plan. To account for clustered data at the clinic level, we will use generalized estimating equations with an independence working correlation structure and a robust sandwich variance estimator and correction for the small number of clinics.

We will also investigate plans to taper medications from the provider and participant perspective. We will capture the providers’ perspective from actions documented in the signetur field. We will display summary statistics indicating whether a taper was documented in the signetur field for each target medication over 6 months following the medication-specific brochure mailing date, and the mean time from brochure mailing date to documented taper plan. To capture the participants’ perspective on possible tapering, we will use information from the returned postcards. We will present summary statistics indicating the number and proportion of response types tabulated by brochure medication class.

For safety outcomes, we will provide the proportion of participants with a safety outcome by group, given that these are likely to be rare events.

### Interim analyses {21b}

No interim analyses are planned for this study.

### Methods for additional analyses (pre-specified subgroup analyses; implementation analyses) {20b}

#### Subgroup analyses

We will conduct subgroup analyses to compare the time to first incident medically treated fall based on the following characteristics: age (< 80 vs 80 +), sex assigned at birth (female vs male), at least one fall prior to the first brochure mailing date, multimorbidity (defined as having 2 or more chronic conditions), and frailty. Subgroup analyses will be performed in a similar manner to the primary analysis: we will consider a cause-specific proportional hazards model, where in addition to the variables included in the primary analysis, we include in the model both the subgroup variable and an interaction between the subgroup variable of interest and assignment to the intervention group. We will estimate the ratio comparing the two subgroup variable values of the adjusted cause-specific hazard ratio comparing intervention and usual care groups and its corresponding 95% confidence interval.

#### Implementation analyses

Three implementation analyses will be conducted. First, we will systematically report adaptations to the original, evidence-based intervention to optimize fit with this particular delivery system and population while maintaining fidelity to the core components of the intervention. Adaptations will be coded according to an established framework for characterizing adaptations of evidence-based practices post implementation to indicate whether an adaptation was made to the context, content, or implementation support [47]. Second, we will describe the host of implementation strategies deployed to disseminate the STOP-FALLS study. Based on reporting recommendations [48] and a pragmatic tracking method, we will code project meetings and supplementary activities to capture detailed information on implementation strategies including the actor of the strategy, the action being performed, the target of the action, temporality, dose, outcome affected, and rationale for strategy selection. We will use structured minute-taking during meetings and notes on implementation activities to capture these data. Finally, we will explore the ways in which clinic-level leaders shaped the form that the intervention took and how well it was integrated into existing workflows. We will assess structured notes from clinic-based presentations and virtual communications with clinic-level leaders, including clinic chiefs and/or clinic champions. Consistent with emerging literature [49], we will classify the degree to which clinic leaders demonstrate proactive, knowledgeable, supportive, and perseverant leadership for the STOP-FALLS intervention. We will analyze the degree to which these influences helped or hindered intervention implementation across the KPWA delivery system comparing leader profiles for low-, moderate-, and high-performing clinics.

### Methods in analysis to handle protocol non-adherence and any statistical methods to handle missing data {20c}

All participants who remained eligible at their initial brochure mailing date will be analyzed according to the group they were originally assigned. Missing data will be closely tracked. If a clinic withdraws, which is very unlikely, we will use a complete-case analysis. Our statistical analysis plan addresses the possibility of participants dropping out of the study through censoring.

### Plans to give access to the full protocol, participant-level data, and statistical code {31c}

A participant-level dataset will not be made available for public release, because a waiver of consent for use of healthcare utilization data was obtained (i.e., study participants did not directly consent to have their data used for research purposes). Statistical code for the analyses of primary and secondary outcomes will be made available to other researchers upon request.

## Oversight and monitoring

### Composition of the coordinating center and trial steering committee {5d}

This study does not have an external coordinating center or trial steering committee. The research team meets twice a week to oversee the progress of the study and completion of tasks. The study leads meet once a month with the study sponsor to review study progress.

### Composition of the data monitoring committee, its role and reporting structure {21a}

With the agreement of the funding agency, a data safety and monitoring board was not appointed due to the low-risk nature of the trial.

### Adverse event reporting and harms {22}

As previously mentioned, medically treated falls, safety events, and deaths are trial outcomes and therefore not reportable to the IRB. The one exception is for safety events related to an ADWE. The study team will monitor the 6-month period following the last intervention mailing for ADWE through chart review; those events determined to be “definite” will be reported to the IRB.

### Frequency and plans for auditing trial conduct {23}

This study is funded under a cooperative agreement with the CDC; as such, the CDC provides auditing for the trial via monthly meetings and an annual site visit.

### Plans for communicating important protocol amendments to relevant parties (e.g., trial participants, ethical committees) {25}

Approval for any study protocol amendments will be obtained from the KPWA IRB which serves as the single IRB for the study. If warranted by the amendment, participant consent materials will be updated accordingly and any changes to the published protocol will be reported in full in any future publications.

### Dissemination plans {31a}

The results from this clinical trial will be fully disclosed via publications in peer-reviewed journals and by oral/poster presentations at national and international scientific meetings. All findings, whether negative or positive, will be reported. We will also report results to KPWA clinical leadership. There is no plan to report results directly to participants.

## Discussion

The STOP-FALLS study builds on evidence demonstrating the effectiveness of direct-to-consumer education on reducing use of potentially inappropriate medications by older adults [22]. The intervention delivers evidence-based guidance on risks of CNS-active medications to both older adults and their PCP. It is a low-cost [50, 51] nudge intervention designed to be replicable by a health system. We hope to demonstrate that the intervention will prompt discussions between patients and their prescribers and ultimately result in a reduction in medically treated falls through tapering and/or discontinuation of CNS-active medications. Engaging the PCP is hypothesized to be central to the mechanism of the intervention, because older adults have a high level of trust in their PCP to provide information about prescription medications [52] and more generally because older patients report that their healthcare provider is their most valued source of health information [53, 54]. STOP-FALLS will be among the first trials designed to assess the effectiveness of deprescribing on a key clinical outcome (i.e., falls).

Several features of the STOP-FALLS study are novel. First, the STOP-FALLS intervention is a modification of an evidence-based deprescribing intervention [20]. The trial testing that intervention, known as D-PRESCRIBE, was conducted in Canada and involved community pharmacies. A central modification thus involved embedding the core components of the intervention in a health system in the USA. Second, STOP-FALLS harnesses the health system’s automated pharmacy data, allowing for identification of patients prescribed one or more of the target medications on a long-term basis. Third, decision support is delivered to PCPs via the EMR using the staff message function. Fourth, delivery of patient-facing intervention materials is timed to coincide with an upcoming primary care visit for a subset of intervention participants. Fifth, the intervention prepares PCPs to address specific challenges around deprescribing through periodic emails containing information regarding risks of targeted medications, treatment alternatives, and communication tips to facilitate conversations with patients around deprescribing.

Study strengths include the pragmatic design, clinic-level randomization that minimizes the risk of contamination, blinding of investigators and data analysts for outcomes assessments, objective assessment of dose reduction/discontinuation using pharmacy fill records and data from signetur fields [33], and targeting multiple classes of CNS-active medications. Other strengths include adaptations of study materials based on input from older adults and primary care providers of the health system in which the trial is being conducted and the intervention’s emphasis on non-pharmacologic alternatives for symptom management. Moreover, the effectiveness-implementation hybrid design allows us to contextualize the main results, and if the intervention proves effective, we can characterize the implementation process for scale up and spread in the KP and other settings.

Study limitations include the setting, which is an integrated delivery system. Results from STOP-FALLS may not be generalizable to other clinical settings. In addition, because pharmacists employed by the delivery system have limited time and availability to engage in research studies, and also because during the time of the trial they have been implementing a system-wide opioid dose reduction initiative, they were unable to take on a dedicated role as part of the intervention, and as a result, we will be unable to assess the effectiveness of systematic involvement of a pharmacist as part of our intervention. Lastly, patient-reported outcomes that may be impacted by the intervention will not be assessed due to the pragmatic nature of trial data collection.

In summary, the STOP-FALLS trial will generate evidence regarding the effectiveness of a health-system-embedded deprescribing intervention on medically treated falls, a patient-centered outcome of high relevance to older adults. Secondary (medication-related) outcomes will illuminate whether a nudge intervention is effective in reducing medications associated with fall risk. Low-intensity interventions that are relatively simple and scalable for health systems are critical for achieving age-friendly care.

## Trial status

The study sample was generated and brochures mailed to the first eligible participants on April 1, 2021. Enrollment closed on June 16, 2022. Participant follow-up will end on June 16, 2023. The study recruitment is completed and at time of this paper submission the study is in the data collection phase. Preparation and submission of the manuscript were delayed due to changes in staff including the site PI. The statistical analysis plan was finalized prior to submission and no outcome data analysis will be conducted until after publication of the study’s protocol.

## Supplementary Information

**Additional file 1:** **Additional File 1.** ICD-10 Fall codes and Injury Indicators for Primary Outcomes.

**Additional file 2:** **Additional File 2.** Method for Creating the Standardized Daily Dose. **eTable 1.** Classification of medications and minimum effective dose (excluding opioids). **eTable 1.** Classification of medications and minimum effective dose (excluding opioids).

## Data Availability

The datasets generated and/or analyzed during the current study will not be made publicly available, as clinical data are the property of the healthcare delivery system and its patient members. Reasonable data requests will be considered by the authors with additional permission of the Kaiser Permanente health care system and its associated Institutional Review Board.

## Abbreviations

ADWE: Adverse drug withdrawal event
CDC: Centers for Disease Control and Prevention
CNS: Central nervous system
EBPO: Evidence-based pharmaceutical opinion
ED: Emergency department
EMR: Electronic medical record
ICC: Intraclass correlation
ICD-10: International Classification of Diseases, Tenth Revision
IGP: Integrated Group Practice
IRB: Institutional Review Board
KPWA: Kaiser Permanente Washington
KPWHRI: Kaiser Permanente Washington Health Research Institute
OTC: Over-the-counter
PCP: Primary care provider
SDD: Standardized daily dose
SIG: Signetur
