# Supplementary material for: A health-system-embedded deprescribing intervention targeting patients and providers to prevent falls in older adults (STOP-FALLS trial): study protocol for a pragmatic cluster-randomized controlled trial
Source: Trials. 2023 May 11;24:322. doi: 10.1186/s13063-023-07336-7 (PMC10173496; doi:10.1186/s13063-023-07336-7)
Supplement: Supplementary file 1 — Additional file 1: Additional File 1. ICD-10 Fall codes and Injury Indicators for Primary Outcomes. [file 13063_2023_7336_MOESM1_ESM.docx]

Additional File 1: ICD-10 Fall codes and Injury Indicators for Primary Outcomes

Codes for Non-fatal Unintentional Fall Related Injury*

| **ICD** | **Code** | **Definition** |
| --- | --- | --- |
|  | V00.11-V00.89 w/ 6^th^ character=1 | Falls related to pedestrian conveyance. The 6^th^ character does not include the decimal point (I.e., the 6^th^ character of “V00.112” is “2”). |
| 10 | W00 | Fall due to ice and snow |
| 10 | W01 | Fall on same level from slipping, tripping and stumbling |
| 10 | W03 | Other fall on same level due to collision with another person |
| 10 | W04 | Fall while being carried or supported by other persons |
| 10 | W05 | Fall from non-moving wheelchair/scooter or motorized mobility scooter |
| 10 | W06 | Fall from bed |
| 10 | W07 | Fall from chair |
| 10 | W08 | Fall from other furniture |
| 10 | W09 | Fall on and from playground equipment |
| 10 | W10 | Fall on and from stairs and steps |
| 10 | W11 | Fall on and from ladder |
| 10 | W12 | Fall on and from scaffolding |
| 10 | W13 | Fall from, out of or through building or structure |
| 10 | W14 | Fall from tree |
| 10 | W15 | Fall from cliff |
| 10 | W16 with 6th character=2 (Except 16.4 and 16.9 with 5th character=2) | Fall, jump, or diving into water |
| 10 | W17 | Other fall from one level to another |
| 10 | W18.1, W18.2, W18.3 | Other slipping, tripping and stumbling and falls |
| 10 | W19 | Unspecified fall |

* 7*77 *7^th^ character of “A” or missing (reflects initial encounter or active treatment)

Injury and certain other consequences of external causes S/T/M diagnosis codes

| **ICD** | **Code** | **Definition** |
| --- | --- | --- |
| 10 | [S00-S09](https://www.icd10data.com/ICD10CM/Codes/S00-T88/S00-S09) | Injuries to the head |
| 10 | [S10-S19](https://www.icd10data.com/ICD10CM/Codes/S00-T88/S10-S19) | Injuries to the neck |
| 10 | [S20-S29](https://www.icd10data.com/ICD10CM/Codes/S00-T88/S20-S29) | Injuries to the thorax |
| 10 | [S30-S39](https://www.icd10data.com/ICD10CM/Codes/S00-T88/S30-S39) | Injuries to the abdomen, lower back, lumbar spine, pelvis and external genitals |
| 10 | [S40-S49](https://www.icd10data.com/ICD10CM/Codes/S00-T88/S40-S49) | Injuries to the shoulder and upper arm |
| 10 | [S50-S59](https://www.icd10data.com/ICD10CM/Codes/S00-T88/S50-S59) | Injuries to the elbow and forearm |
| 10 | [S60-S69](https://www.icd10data.com/ICD10CM/Codes/S00-T88/S60-S69) | Injuries to the wrist, hand and fingers |
| 10 | [S70-S79](https://www.icd10data.com/ICD10CM/Codes/S00-T88/S70-S79) | Injuries to the hip and thigh |
| 10 | [S80-S89](https://www.icd10data.com/ICD10CM/Codes/S00-T88/S80-S89) | Injuries to the knee and lower leg |
| 10 | [S90-S99](https://www.icd10data.com/ICD10CM/Codes/S00-T88/S90-S99) | Injuries to the ankle and foot |
| 10 | T07-T34 | Foreign bodies, burns, corrosions, frostbite |
| 10 | T36-T50 with a 6^th^ character of 1,2,3,4. Except: T36.9, T37.9, T39.9, T41.4, T42.7, T43.9, T45.9, T47.9, and T49.9 with a 5^th^ character of 1,2,3,or 4 | Drug poisoning – excludes adverse events and under dosing |
| 10 | T51-T65 | Toxic effects of substances nonmedicinal as to source |
| 10 | T66-T76 | Other and unspecified effects of external causes. |
| 10 | [T79](https://www.icd10data.com/ICD10CM/Codes/S00-T88/T79-T79) | Certain early complications of trauma |
| 10 | M97 | Peri prosthetic fracture around internal prosthetic joint |
| 10 | V00.11-V00.89 w/ 6^th^ character=18 | Falls related to pedestrian conveyance |

* 7^th^ character of “A”, “B”, or “C”, or missing reflects initial encounter/ active treatment. T30-32 do not have a 7^th^ character.
