# Supplementary material for: A health-system-embedded deprescribing intervention targeting patients and providers to prevent falls in older adults (STOP-FALLS trial): study protocol for a pragmatic cluster-randomized controlled trial
Source: Trials. 2023 May 11;24:322. doi: 10.1186/s13063-023-07336-7 (PMC10173496; doi:10.1186/s13063-023-07336-7)
Supplement: Supplementary file 2 — Additional file 2: Additional File 2. Method for Creating the Standardized Daily Dose. eTable 1. Classification of medications and minimum effective dose (excluding opioids). eTable 1. Classification of medications and minimum effective dose (excluding opioids). [file 13063_2023_7336_MOESM2_ESM.docx]

**Additional File 2: Method for Creating the Standardized Daily Dose**

1. Standardized daily dose (SDD) of target medications will be calculated as follows:
   - We first calculate the daily dose for each target medication prescription by multiplying the medication strength by the number of tablets/days supply.
   - We then calculate the SDD by dividing the daily dose (Column A) by the minimum effective geriatric daily dose for that specific medication as reported in a commonly used geriatric pharmacotherapy source (Column B)^1^ (**see eTable** **1**).

Case example:

| **From Pharmacy Fill Data** | | | **Column A** | **Column B** |  |
| --- | --- | --- | --- | --- | --- |
| Medication, strength | Number of tablets | Days supply | Daily Dose | Minimum effective geriatric daily dose | SDD  (colA/Col B) |
| Zolpidem 10 mg | 30 | 30 | (10 x 30)/30=10 | 5 mg | 2 |
|  |  |  |  |  |  |

1. Semla TP, Beizer JL, Higbee MD. Geriatric Dosage Handbook. Hudson, OH: Lexicomp; 2011.

**eTable 1: Classification of medications and minimum effective dose (excluding opioids).**

| **Drug Class** | **Individual Medications** | **Minimum Effective Geriatric Daily Dose** |
| --- | --- | --- |
| **Benzodiazepines/Sedative Hypnotics** | | |
|  | Alprazolam | 0.25 mg |
|  | Chlordiazepoxide | 10 mg |
|  | Clonazepam | 0.5 mg |
|  | Clorazepate | 7.5 mg |
|  | Diazepam | 1 mg |
|  | Eszopiclone | 1 mg |
|  | Flurazepam | 15 mg |
|  | Lorazepam | 0.5 mg |
|  | Oxazepam | 20 mg |
|  | Temazepam | 7.5 mg |
|  | Triazolam | 0.0625 mg |
|  | Zaleplon | 5 mg |
|  | Zolpidem | 5 mg |
|  | Zolpidem extended release | 6.35 mg |
|  | | |
| **Tricyclic antidepressants** | Nortriptyline | 10 mg |
|  | Amoxapine | 25 mg |
|  | Trimipramine | 50 mg |
|  | Maprotoline | 25 mg |
|  | Protriptyline | 15 mg |
|  | Desipramine | 10 mg |
|  | Clomipramine | 25 mg |
|  | Amitriptyline | 10 mg |
|  | Amitriptyline/Perphenazine | 10 mg |
|  | Doxepin | 10 mg |
|  | Imipramine | 10 mg |
|  | Nortriptyline | 10 mg |
| **Skeletal muscle relaxants** |  |  |
|  | Baclofen | 5 mg |
|  | Carisoprodol | 250 mg |
|  | Cyclobenzaprine | 5 mg |
|  | Dantrolene | 25 mg |
|  | Methocarbamol | 500 mg |
|  | Tizanidine hydrochloride | 2 mg |
|  |  |  |
| **Antihistamines** | Brompheniramine | 12 mg |
|  | Carbinoxamine | 6 mg |
|  | Chlorpheniramine | 4 mg |
|  | Clemastine | 2 mg |
|  | Cyproheptadine | 4 mg |
|  | Dexbrompheniramine | 4 mg |
|  | Dexchlorpheniramine | 4 mg |
|  | Dimenhydrinate | 200 mg |
|  | Diphenhydramine | 50 mg |
|  | Doxylamine | 5 mg |
|  | Hydroxyzine | 75 mg |
|  | Meclizine | 25 mg |
|  | Promethazine | 50 mg |
|  | Pyrilamine | 30 mg |
|  | Triprolidine | 10 mg |

**e.Table 2: Oral Morphine Equivalents/Conversions Used for Study Opioids**

**To create SDD for opioids we use a slightly different process**

- Multiply total daily dose of opioid by the conversion factor (3^rd^ column) and divide by 10mg/day (minimum effective geriatric dose for morphine)

| **Opioid** | **Formulation** | **Oral Morphine Equivalent Conversion Factor (30 mg)*** |
| --- | --- | --- |
| **Codeine** | Oral | 0.15 |
| **Buprenorhpine** | Transdermal  (7-day patch) | 1.8 |
| **Fentanyl** | Transdermal  (3-day patch) | 2.4 |
| **Hydrocodone** | Oral | 1 |
| **Hydromorphone** | Oral | 5 |
| **Methadone** | Oral | 4.7 |
| **Morphine** | Oral | 1 |
| **Oxycodone** | Oral | 1.5 |
| **Tramadol** | Oral | 0.2 |
| **Tapentadol** | Oral | 0.4 |
